# Changes in the risk management of *Salmonella enterica* subspecies *diarizonae* serovar 61:(k):1, 5, (7) in Swedish sheep herds and sheep meat due to the results of a prevalence study 2012

**DOI:** 10.1186/s13028-015-0096-0

**Published:** 2015-02-03

**Authors:** Kaisa Sörén, Mats Lindblad, Cecilia Jernberg, Erik Eriksson, Lennart Melin, Helene Wahlström, Maria Lundh

**Affiliations:** National Veterinary Institute, SE-751 89 Uppsala, Sweden; National Food Agency, P.O. Box 622, SE-751 26 Uppsala, Sweden; The Public Health Agency of Sweden (previously the Swedish Institute for Communicable Disease Control), SE-171 82 Solna, Sweden; The Swedish Board of Agriculture, SE-551 82 Jönköping, Sweden

**Keywords:** *Salmonella diarizonae* 61:(k):1, 5, (7), Prevalence, Sheep, Sweden, Risk management, *Salmonella* control

## Abstract

**Background:**

The prevalence of *Salmonella* in food producing animals is very low in Sweden due to rigorous control programmes. However, no active surveillance is in place in sheep. The authorities decided to perform a prevalence study in sheep herds because findings at slaughter indicated that sheep associated *S. diarizonae* (*S. enterica* subspecies *diarizonae* serovar 61:(k):1, 5, (7)) might be common in sheep. Sampling was stratified by herd size in two groups, small herds with ≤ 30 animals and large herds with > 30 animals. In each stratum, 237 herds were selected at random. Faecal samples received from 244 out of the 474 randomly selected herds were analysed.

**Results:**

A total of 40 of 100 (40%) of large herds and 17 of 144 (12%) of small herds were positive. The overall adjusted prevalence was 17.6% (95% CI, 12.9-22.2). Sheep associated *S. diarizonae* was detected in all counties (*n* = 21). Scientific opinions and an evaluation of on-farm control measures performed concluded that the impact of sheep associated *S. diarizonae* on human health is very low, and that risk management measures applied in response to findings of sheep associated *S. diarizonae* in sheep or sheep meat can be expected to have very little impact on reducing risks to human health. As a result, Swedish authorities decided to make an exemption for sheep associated *Salmonella diarizonae* in sheep and sheep meat in the current *Salmonella* control measures.

**Conclusions:**

Sheep associated *S. diarizonae* is endemic in Swedish sheep herds. It is more common in large herds and not limited to certain parts of the country. The responsible authorities concluded that current risk management actions regarding sheep associated *S. diarizonae* in sheep and sheep meat are not proportional to the risk. This is the first time in the history of the Swedish *Salmonella* control programme that an exemption from the legislation has been made for a specific serovar. If there is any future indication of an increasing risk, due to *e.g.* change in the pathogenicity or development of antimicrobial resistance, the risk assessment will be re-evaluated and control measures reinforced if needed.

## Background

Bacteria of the species *Salmonella enterica* occur world-wide and are a common cause of gastro-intestinal infections in both humans and animals. Bacteria belonging to *S. enterica* are divided into six subspecies consisting of more than 2,500 different serovars [1]. Among these, *S. enterica* subspecies *diarizonae* serovar 61:(k):1, 5, (7) (sheep associated *S. diarizonae* (SASd)) is considered as adapted to sheep [2-4]. The bacteria have been reported to be commonly found in sheep in the United Kingdom (UK), Norway and Switzerland [5-8]. The infection in sheep is usually sub-clinical [9] but may cause enteritis, rhinitis, orchitis, and aborted or stillborn foetuses [5,10,11]. SASd is only occasionally isolated from other species [12] and seldom reported in humans. For example, in the UK two human cases were reported during the time period 1966–1990, in the USA, 27 cases were reported between the years 1967–1976 and in Norway, only one case has been reported [4,13,14]. The situation is similar in Sweden, where only one case, which was travel related, has been reported during the last 25 years. *S. diarizonae* may have atypical growth characteristics, presenting difficulties in identifying this subspecies if laboratory personnel do not have experience with it. The underdiagnosis of this subspecies may therefore be larger compared to *S. enterica* subspecies *enterica*.

In Sweden, as in Norway and Finland, the prevalence of *Salmonella* in food producing animals is very low due to a control programme initiated more than 50 years ago [15]. Any finding of *Salmonella* in feed, animals and food is notifiable and actions are always taken to eliminate the infection/contamination. Infected herds, including sheep herds, are put under restrictions and live animal movements are prohibited. Measures to improve the hygiene, cleaning and disinfection of the stable environment, and if necessary the feeding system and other contaminated areas, and when relevant, elimination of chronically infected animals, are used to eliminate the infection. Two consecutive whole-herd samplings with negative results are required to consider a herd free from infection and lift restrictions [16]. In sheep, 50% of the costs of eradication are funded by the Board of Agriculture. Fresh meat from carcasses of any animal species with demonstrated presence of *Salmonella* is not considered safe for human consumption and has to be placed on the market for a meat product plant or destroyed.

In contrast to cattle, swine and poultry, there is no active surveillance for *Salmonella* in sheep. Instead, the surveillance relies on passive surveillance including post mortem examinations. However, because infection with SASd is typically subclinical in sheep [9], this surveillance is expected to have a low sensitivity. Clinical disease was only identified in 3 of 11 SASd-infected sheep-herds identified between 1998 and 2010 [17]. In none of these cases was SASd considered to be the cause of the observed disease. This indicates that active surveillance is needed to detect SASd-infected sheep herds. In 1998, a slaughterhouse survey showed that 3 out of 605 (0.5%) faecal samples from ewes and 2 out of 404 (0.5%) faecal samples from lambs were positive for SASd. No other *Salmonella* types were found [18]. In 2006, swab sampling of sheep carcasses at slaughterhouses according to Commission Regulation (EC) No 2073/2005 commenced. SASd-contaminated sheep carcasses have been detected in this sampling, indicating that the prevalence of *Salmonella* in sheep might be higher compared to other food producing animals. However, due to lack of denominator data, the prevalence of positive sheep carcasses could not be estimated. No studies have been performed to assess the prevalence of SASd at the herd level.

In Norway, SASd is considered to be endemic, with an overall herd prevalence of 12% but with uneven geographical distribution, the regional prevalence varying from 0 to 45% [6]. Norway has a *Salmonella* control programme as Sweden, but based on a risk assessment that concluded that the impact of SASd on human health in Norway appeared to be marginal [19], control measures taken at the herd-level have been changed in Norway and actions are restricted to herds with clinical illness. It has not yet been decided which risk management actions will be taken when SASd is isolated from fresh meat from sheep carcasses in Norway (Kjell Hauge, personal communication, 2013).

There were indications that the prevalence of SASd might also be high in Sweden, indicating that the present control programme was not efficient. Furthermore, there were no indications that this serovar was commonly reported in humans. If this was the case, the benefits of the present control on *Salmonella* in sheep could be questioned. The relevant authorities jointly decided to perform a prevalence study in sheep herds. Based on the results from the study, the authorities would further assess how to manage this *Salmonella* type when isolated from sheep. The aim of the present paper is to describe the prevalence study conducted during the winter 2012 and the actions taken by authorities due to the result of the study.

## Methods

### Study design

The National Veterinary Institute (SVA) obtained data on sheep herds (*n* = 16,478) from the Board of Agriculture. After exclusion of 1,657 herds where data on herd size was missing, 14,821 herds remained. Altogether these herds contained 409,181 sheep. The herd size distribution was skewed, 79.9% of the herds were considered small with 30 sheep or less and 20.1% had between 31 and 1,425 sheep. The aim was to detect an among-herd prevalence of 1% with 95% confidence. Given a herd sensitivity of 95% (as detailed below), this required that 315 herds were sampled [20]. As it was expected that the number of non-responders might be high, the sample size was increased by 50% to 474 herds. As the costs for eradication of *Salmonella* from herds are much higher in large herds we wanted to avoid that most testing would be done in small herds. Sampling was therefore stratified by herd size in 2 groups, small herds with ≤30 sheep and large herds with >30 sheep. In each stratum, 237 herds were selected at random.

In each herd enough samples were to be collected to produce a 95% herd sensitivity assuming that in a positive herd, 10% of adult sheep (>1 year) excrete sufficient amount of bacteria to be detected by bacteriological culture [19]. Due to practical and economical reasons it was decided to pool 15 samples in one pool. It was assumed that 50% of the sheep in a herd were adult (Kalle Hammarberg, personal communication, 2011), i.e. small herds were herds assumed to have ≤15 adult sheep and large herds >15 adult sheep. Assuming a test sensitivity of 1, 1 pooled sample was needed to detect a prevalence of 10% with 95% confidence level in herds with up to 19 adult sheep [20] and in larger herds 2 pooled samples were needed.

### Sampling

The sampling was done by the animal owners and the study was conducted anonymously so that fear of control measures would not prevent farmers from participating. Every selected animal owner received a sampling-kit in February 2012 consisting of plastic gloves, 1 or 2 plastic sample collection containers, a padded envelope and a letter with instructions on how to take the sample(s). A referral note to be sent back to the laboratory with the sample(s), where the number of sheep in the herd excluding lambs born in 2012 was to be noted, was included. The county in which the herd was situated was written on the note before it was sent out to the animal owner.

The animal owner was instructed to pick a total amount of forty fresh round pieces of faeces (corresponding to at least 25 g faeces) from adult animals from the bedding for 1 pooled sample. Faeces were to be picked from at least 15 different places, in order to represent at least 15 different animals, and from different parts of the herd if the animals were kept in several buildings or pastures. The faeces were collected in the plastic sample collection container(s) and posted to the SVA together with the referral note. The only information about the participating herds that the SVA had access to was the number of animals in the herds and the counties in which the herds were situated.

### Analysis of faecal samples

Analysis of faecal samples was done at the SVA. In case a pooled sample weighted more than 25 g, faeces corresponding to 25 g were picked from different parts of the sample. The 25 g samples were then analysed for presence of *Salmonella* using the MSRV enrichment method (ISO 6579:2002/Amd 1:2007 Annex D), and each isolate was typed biochemically and sero-typed by agglutination of O-antigen and flagellar antigen according to the White-Kauffman-Le Minor scheme [1], in order to confirm if it was SASd.

### Statistical analysis

The herd-size distribution as well as average herd sizes in small and large herds in source population and study sample were compared. The overall herd prevalence of SASd was calculated by weighting the prevalence in small herds (representing 79.9% of the population), and large herds (representing 20.1% of the population),

$$ {p}_h = 0.799x{p}_{sh} + 0.201x{p}_{lh} $$

where *p*_*h*_ is the herd prevalence and *p*_*sh*_ and *p*_*lh*_ are the prevalences in small and large herds respectively. The overall 95% confidence interval was calculated as 1.96 √ σ^2^, using a pooled variance σ^2^,

$$ {\sigma}^2 = pro{p_{sh}}^2x\left({p}_{sh}\left(1-{p}_{sh}\right)\right)/{n}_{sh}+ pro{p_{lh}}^2x\left({p}_{lh}\left(1-{p}_{lh}\right)\right)/{n}_{lh} $$

where *prop*_*sh*_ and *prop*_*lh*_ are the proportions of small and large herds in the population respectively and *p*_*sh*_ and *p*_*lh*_ are the prevalence of *Salmonella* in small and large herds respectively. The values *n*_*sh*_ and *n*_*lh*_ are the number of small and large herds that were tested, respectively. To evaluate the effect of herd size on the probability of being infected, herds were divided into 5 categories that were biologically reasonable (≤15, 16–30, 31–60, 61–100, >100 adult sheep) and the prevalence and 95% confidence interval were calculated.

### Scientific opinions and evaluation of on-farm control measures

Due to the result of the prevalence study, the National Food Agency (NFA) delivered a scientific opinion on the extent of the risk that SASd in sheep may apose to public health. Specific questions included: to what degree SASd can be considered to be pathogenic to humans, and whether there is a basis that may justify a different risk management of this serovar compared to other *Salmonella* types in Sweden. To further investigate the prevalence of SASd in the food chain, data on swab samples from sheep carcasses from one of the largest sheep slaughterhouses in Sweden were collected. In total, 990 swab samples were taken by the company 2007 – 2011. The sampling was performed according to the requirements in Commission Regulation (EC) No 2073/2005. In addition, the Swedish Institute for Communicable Disease Control (SMI) was requested by the Board of Agriculture and the NFA to deliver a scientific opinion on the impact of SASd on human health.

The Board of Agriculture initiated an evaluation of on-farm control measures for SASd. The consequences of on-farm control measures as practiced were compared with the expected consequences of alternative options, such as refraining from control measures or eradication of SASd from the sheep population. The most critical question was how a change of on-farm control measures would be expected to affect public health. In addition, the costs of control measures were investigated. Calculations were made for two scenarios; one representing the current level of detection, and one simulating hypothetical detection of all infected herds. Costs for farmers as well as for the state were taken into account. The potential effects on animal health and antimicrobial resistance were also considered.

## Results

### Prevalence study

Samples were received from a total of 262 (55%) out of the 474 randomly selected herds. Eighteen herds were excluded for the following reasons: *i*) missing herd size (*n* = 10), *ii*) 1 pooled sample instead of 2 was collected (*n* = 7) and *iii*) 2 samples instead of 1 were collected (*n* = 1). A total of 244 (51%) herds remained for further analysis. Of these herds, 144 were small herds and 100 were large. The herd size distribution of the source population and the study sample in the 2 groups was similar. The average herd size of small herds was 13.5 in the source population and 9.8 in the study sample. The average herd size of large herds was 75.6 in the source population versus 94.6 in the study sample. A total of 40 of 100 (40%) of the large herds and 17 of 144 (12%) of the small herds were positive for SASd. The overall adjusted prevalence was 17.6% (95% CI, 12.9 – 22.2). No other *Salmonella* type was found. The proportion of positive herds increased with herd size (≤15, 16–30, 31–60, 61–100, >100 adult sheep) from 0.06, 0.22, 0.26, 0.54 to 0.61. The 95% confidence intervals were wide and overlapped between groups. Positive herds were found in all 21 counties in Sweden.

### Scientific opinion by the NFA

The NFA opinion concluded that the present prevalence study indicated that SASd is common in Swedish sheep herds, as is also the case in other countries, *e.g.* United Kingdom, Norway and Switzerland [5-8]. Data on swab samples from sheep carcasses from one of the largest sheep slaughterhouses in Sweden showed that 18 (1.8%) of the 990 swab samples taken between 2007 and 2011 were positive for *Salmonella* (all identified as SASd). This is a significantly higher prevalence of *Salmonella* than for cattle and swine carcasses. During the same time period (2007 to 2011) only 0.03% of 16,928 carcass swab samples from cattle and 0.02% of 29,583 carcass swab samples from swine were *Salmonella* spp. positive [21]. Assuming that the prevalence of SASd at this slaughterhouse is representative of all sheep slaughtered in Sweden, about 4,700 sheep carcasses would be expected to be contaminated with SASd each year. The corresponding figures for presence of *Salmonella* spp. on cattle and swine are 100 and 700 carcasses, respectively.

In summary, SASd was considered to have low virulence in humans because the number of reported human cases in Sweden and other countries where this serovar is common in sheep and sheep carcasses is very low, despite the fact that consumers are likely exposed to it in relatively high extent by sheep meat. Consequently, the significance of SASd for public health was assessed to be significantly lower than that of serovars belonging to *S. enterica* subspecies *enterica*. It was concluded that risk management measures applied at findings of SASd in sheep or sheep meat can be expected to have very little impact on reducing risks to human health.

### Scientific opinion by the Swedish Institute for Communicable Disease Control

In the scientific opinion of SMI, the impact of SASd on human health was considered when taking the potential underdiagnosis of *S. diarizonae* into account. Facts taken into consideration included the following: *i*) there were a low number of reported human cases of SASd in Sweden, *ii*) there was no invasive human infection with SASd reported in Sweden and *iii*) the majority of the human clinical laboratories in Sweden had reported *S. diarizonae*, mostly from faecal samples, i.e. they were able to isolate this subspecies from faeces which is the most complex material to isolate from. Furthermore, a blind test including one typical and one atypical *S. diarizone* was conducted at a clinical laboratory and the test results were correct. This was a limited test, however, this primary hospital laboratory was used for consultation regarding the possibility of any isolation difficulties regarding SASd. Based on these facts it was concluded that SASd has limited impact on human health.

### Evaluation of on-farm control measures by the Board of Agriculture

The primary consideration for evaluating control measures on-farm were the opinions of the NFA and SMI regarding the effects on public health. In addition, the Norwegian risk assessment [19] was taken into account. The prevalence study indicated that approximately 2,720 SASd-infected sheep herds existed in Sweden, but during the last five years (2008–2012) only 1–2 infected herds were detected per year. Thus, the number of unknown infected herds was substantial. During the years 2008 – 2011, the average cost for control measures in a SASd-infected herd was 29,000 € (range 6,800 – 55,900 €), divided evenly between the state and the producer. If all infected herds in Sweden could hypothetically be detected, the costs of eradication were estimated to be at least 84 million € shared between producers and the state. Data based on necropsy statistics and health-monitoring did not indicate that SASd had any considerable impact on animal health in Sweden. Since monitoring of antimicrobial resistance in the Swedish Veterinary Antimicrobial Resistance Monitoring programme started at the SVA in 2000, isolates of *S. diarizonae* from 9 separate incidents in sheep have been tested and resistance has not been found in any of the isolates [22]. The Board of Agriculture concluded that the sensitivity of the present surveillance is very low and that measures taken in identified infected herds have practically no effect on public health and probably no effect on the prevalence of SASd in the larger sheep population. On the other hand, the impact of risk management actions on the single farmer’s economy is significant. To continue on-farm control measures or to try to eradicate SASd from the sheep population would be very expensive and, as shown in the scientific opinions of the NFA and the SMI, would be expected to have little gain to public health.

### Risk management

Based on the abovementioned opinions and the evaluation of on-farm control measures, the Board of Agriculture and NFA decided to change national regulations concerning control measures regarding SASd in sheep. No changes to the existing control measures will be made for SASd in other animal species. Control measures on-farm will no longer be taken if SASd is isolated in a sheep herd. However, despite the change, the Board of Agriculture will still have the possibility to take action on individual cases. Furthermore, if change in the pathogenicity or development of resistance would occur, the exemption for SASd will be reconsidered. All findings of SASd in sheep will still be notifiable and all isolates will be tested for resistance to antibiotics.

The NFA has made an exemption in the national legislation for findings of SASd on sheep carcasses, i.e. fresh meat from sheep carcasses contaminated with this serovar is not considered unsafe for human consumption. The exemption applies only to fresh meat from sheep carcasses and not fresh meat from cattle, pigs and poultry. Whether any risk management actions will be taken at findings of this serovar in fresh sheep meat at the retail level will be dependent on the responsible local authorities, who could consider the meat unsafe in accordance with Commission Regulation (EC) No 178/2002. Furthermore, due to EU-legislation (Commission Regulation (EC) No 2073/2005) minced meat, meat preparations or mechanically separated meat from any species contaminated with SASd is considered unsafe for human consumption.

Trace back investigations from human cases will continue, i.e. if a sheep herd is a potential source of infection, bacteriological examination of the herd will be done to verify the source of infection. However, the general practices will be that no control measures will be taken in the source herd.

## Discussion

The responsible authorities concluded that current risk management actions regarding SASd in sheep and sheep meat are not proportional to the risk. By making exemptions for SASd in sheep in national legislation regarding on-farm control of *Salmonella*, the annual costs for control of this serovar in sheep herds can be avoided without any adverse effect on human health. The effect of the change in risk management of fresh meat from sheep carcasses at slaughterhouses is much more limited, since only a few SASd-positive carcasses per year are expected to be found. However, the administrative and actual costs for handling positive findings in terms of contacts with authorities and animal owners, withdrawal when products are placed on the market or destruction of carcasses, and extra hygienic measures at slaughterhouses after positive findings, are expected to decrease. Costs for monitoring of sheep carcasses at slaughterhouses according to the requirements in Commission Regulation (EC) No 2073/2005 still remain. Concerning findings of SASd in cattle, pigs and poultry no changes will be made since these species are included in the EU-approved Swedish *Salmonella* control program, making any immediate changes difficult to carry through. Also, for these species findings of SASd are very rare and thus do not result in great costs. The exemption of SASd from regular control measures in Sweden, including both sheep and sheep meat, will be more extensive than in Norway where exemptions currently only concern live animals. Presently, Norway is also considering to exclude SASd from regular control measures when isolated from sheep meat, both at slaughterhouses, cutting plants and at retail level (Kjell Hauge, personal communication, 2013).

The majority of the human clinical laboratories in Sweden use the reference methodology [23] when analysing *Salmonella* in clinical samples. Some strains of SASd may show atypical growth characteristics, i.e. colony morphologies may differ from what is expected for *Salmonella* spp. The colonies may be small and may also, due to the lactose fermenting capacity of SASd, exhibit the ‘wrong’ colour on selective agars [24]. To further evaluate the possibility of underdiagnosis, SASd was included in the annual External Quality Panel (EQA) on feacal diagnostics in the fall of 2012, in which the majority of the clinical laboratories in Sweden are enrolled. The purpose of that specific panel was to investigate the laboratories’ ability to identify *Salmonella* with atypical growth characteristics. All clinical laboratories (*n* = 24) were able to identify SASd as a *Salmonella* (data not shown) (information kindly provided by Equalis, personal communication: results from the EQA program in faecal diagnostics, 2012–38).This supports the conclusion from the scientific opinion of SMI that the low occurence of SASd in humans in Sweden is not due to underdiagnosis.

The prevalence study showed that the apparent prevalence of SASd in sheep herds in Sweden is 17.6% (95% CI, 12.9 – 22.2). As the sensitivity of the test used is not known the true prevalence cannot be calculated but it is assumed to be slightly higher. The among-herd *Salmonella* prevalence in sheep is much higher than the *Salmonella* prevalence in other food producing animals. The prevalence of infected sheep herds in Sweden is in the same range as in Norway, however in contrast to Norway [6] no difference in geographical distribution of positive herds could be found in Sweden. The probability of a herd being positive increased with herd size. This is in agreement with previous studies in Norway [6,25], and seems reasonable because in small herds, the probability of becoming infected is expected to be smaller and the probability of spontaneous elimination of the infection may be higher. Therefore, if average herd size continues to increase in Sweden, the proportion of infected herds may also increase. However, even if this does occur, the risk for human infections is still considered to be negligible.

Historically the Swedish *Salmonella* control programme has covered all serovars, thereby preventing introduction and spread of serovars such as *S*. Enteritidis and multiresistant *S*. Typhimurium. This is the first time an exemption from the legislation has been made for a specific serovar. At present it cannot be foreseen that requirements of control measures would cease for any other serovar of *Salmonella*. Furthermore, if there is any future indication of an increasing risk for humans the present risk assessment will be re-evaluated and control measures reinforced if needed. It is therefore important to continuously monitor any changes in the situation such as changes in the number of human cases, as well as in antibiotic susceptibility of the bacteria. Although an increased prevalence in sheep is not considered to be a potential risk for humans, continued monitoring/surveillance of this serovar in sheep and other animals is also essential.

## Conclusions

The results of the study showed that SASd is endemic in Swedish sheep herds. It is more common in large herds and not limited to certain parts of the country. The responsible authorities concluded that current risk management actions regarding SASd in sheep and sheep meat are not proportional to the risk. This is the first time in the history of the Swedish *Salmonella* control programme that an exemption from the legislation has been made for a specific serovar. If there is any future indication of an increasing risk, due to *e.g.* change in the pathogenicity or development of antimicrobial resistance, the risk assessment will be re-evaluated and control measures reinforced if needed.

## References

[1] Grimont PAD, Weill FX: Antigenic formulae of the Salmonella serovars, 9^th^ edition. WHO Collaborating Centre for Reference and Research on Salmonella. 2007.

[2] Harvey RWS, Price TH, Dixon JMS (1966). Salmonellas subgenus III (Arizona) isolated from abattoirs in England and Wales. J Hyg (Cam).

[3] Martin WJ, Fife MA, Ewing HW (1967). The occurrence and distribution of the serotypes of Arizona. Report of the Center for Disease Control.

[4] Hall ML, Rowe B (1992). *Salmonella arizonae* in the United Kingdom from 1966 to 1990. Epidemiol Infect.

[5] Davies RH, Evans SJ, Preece BE, Chappell S, Kidd S, Jones YE (2001). Increase in Salmonella enterica subspecies diarizonae serovar 61:k:1,5, (7) in sheep. Vet Rec.

[6] Alvseike O, Skjerve E (2002). Prevalence of a *Salmonella* subspecies *diarizonae* in Norwegian sheep herds. Prev Vet Med.

[7] Zweifel C, Zychowska MA, Stephan R (2004). Prevalence and characteristics of Shiga toxin-producing *Escherichia coli*, *Salmonella* spp. and *Campylobacter* spp. isolated from slaughtered sheep in Switzerland. Int J Food Microbiol.

[8] Bonke R, Wacheck S, Bumann C, Thum C, Stüber E, König M (2012). High prevalence of *Salmonella enterica* subsp. *diarizonae* in tonsils of sheep at slaughter. Food Res Int.

[9] Pritchard J (1990). *Salmonella arizonae* in sheep. Can Vet J.

[10] Ferreras MC, Muñoz M, Pérez V, Benavides J, García-Pariente C, Fuertes M (2007). Unilateral orchitis and epididymitis caused by *Salmonella enterica* subspecies *diarizonae* infection in a ram. J Vet Diagn Invest.

[11] Lacasta D, Ferrer LM, Ramos JJ, Bueso JP, Borobia M, Ruiz de Arcaute M (2012). Chronic proliferative rhinitis associated with *Salmonella enterica* subspecies *diarizonae* serovar 61:k:1, 5, (7) in sheep in Spain. J Comp Pathol.

[12] Anonymous (1999). Salmonella in livestock production 1998.

[13] Alvseike O, Vardund T, Lindstedt B, Heir E, Eriksson E, Kapperud G (2004). Molecular epidemiology and population genetics of *Salmonella* subspecies *diarizonae* in sheep in Norway and Sweden. Epidemiol Infect.

[14] Weiss SH, Blaser MJ, Paleologo FP, Black RE, Mc Whorter AC, Asbury MA (1986). Occurrence and distribution of serotypes of the Arizona subgroup of Salmonella strains in the United States from 1967 to 1976. J Clin Microbiol.

[15] National Veterinary Institute. Surveillance of infectious diseases in animals and humans in Sweden 2012. [http://www.sva.se/upload/Redesign2011/Pdf/Om_SVA/publikationer/Surveillance2012.pdf] (accessed on 7 October 2013).

[16] National Veterinary Institute. [http://www.sva.se/en/Animal-health/Zoonoses/Salmonella-as-a-zoonosis/ControlSurveillance/] (accessed on 7 October 2013).

[17] Sternberg Lewerin S, Elvander M (2012). *Salmonella diarizonae* i svenska fårbesättningar. Svensk Vettidn.

[18] Engvall A (1998). Prevalensstudien – får zoonotiska patogen vid slakt hösten 1998.

[19] The Norwegian Scientific Committee for Food Safety (VKM). *Salmonella diarizonae* hos dyr i Norge – konsekvenser for dyr och mennesker. 2008; ISBN:978-82-8082-265-9.

[20] AusVet Animal Health Service. Epitools epidemiological calculators. [http://www.ausvet.com.au/]. (accessed 16 January 2012).

[21] National Veterinary Institute. [http://www.sva.se/en/Animal-health/Zoonoses/Salmonella-as-a-zoonosis/Statistics/] (accessed on 7 October 2013).

[22] National Veterinary Institute. Swedish Veterinary Antimicrobial Resistance Monitoring. [http://www.sva.se/sv/Mer-om-SVA1/Publikationer/Antibiotikaresistens/?lid=32744] (accessed on 7 October 2013).

[23] Public Health Agency of Sweden. [http://referensmetodik.folkhalsomyndigheten.se/w/Salmonella-laboratoriediagnostik] (accessed on 2 April 2014).

[24] Alvseike O, Skjerve E (2000). Probability of detection of Salmonella using different analytical procedures, with emphasis on subspecies diarizonae serovar 61:k:1, 5, (7) [S. IIIb 61:k:1, 5, (7)]. Int J Food Microbiol.

[25] Sandberg M, Alvseike O, Skjerve E (2002). The prevalence and dynamics of *Salmonella enterica* IIIb 61:k:1, 5, (7) in sheep flocks in Norway. Prev Vet Med.

